# Resident Physician Wellness Curriculum: A Study of Efficacy and Satisfaction

**DOI:** 10.7759/cureus.5314

**Published:** 2019-08-03

**Authors:** Dennis Lefebvre, Kathryn A Dong, Erica Dance, Rhonda J Rosychuk, Mark Yarema, Danielle Blouin, Jennifer Williams, Brian H Rowe

**Affiliations:** 1 Emergency Medicine, University of Alberta, Edmonton, CAN; 2 Emergency Medicine, Addiction Medicine, University of Alberta, Edmonton, CAN; 3 Pediatrics, University of Alberta, Edmonton, CAN; 4 Emergency Medicine, University of Calgary, Calgary, CAN; 5 Emergency Medicine, Kingston Health Sciences Centre / Queen's University, Kingston, CAN; 6 Emergency Medicine, University of British Columbia, Kelowna, CAN; 7 Emergency Medicine, School of Community Based Medicine, University of Alberta, Edmonton, CAN

**Keywords:** wellness, physician well-being, physician burnout, resident wellness, resident training, resident curriculum

## Abstract

Background

Recent literature highlights the alarming prevalence of burnout, depression, and illness during residency training; a trend that is also linked to suboptimal patient care. Dedicated wellness curricula may be one solution to this concerning issue.

Purpose

To determine the effect of a multi-faceted wellness curriculum during emergency medicine residency training on wellness scores and to assess resident satisfaction with the program.

Methods

This study was conducted via a longitudinal survey. In 2009, a faculty-derived resident wellness curriculum (F-RWC) was initiated. This program was then bolstered with a parallel resident-derived curriculum (R-RWC) one year later, in 2010. Emergency medicine residents were surveyed in 2009, 2010, and 2011 to assess wellness at baseline, after one year of the F-RWC, and after one year of combined RWCs, respectively. Surveys included two validated assessment instruments (the Brief Resident Wellness Profile (BRWP) and the SF-8^TM^ Health Survey), a satisfaction Likert scale, and a demographics information sheet.

Results

The survey response rates were 89% (n=17), 100% (n=17), and 83% (n=24) from 2009, 2010, and 2011, respectively, for a total of 58 participants. From baseline in 2009, there was a significant improvement in resident wellness, with the addition of parallel RWC by 2011, as measured by the BRWP (p=0.024). The faces scale, a subset of the BRWP, showed a trend toward benefit but did not reach statistical significance (p=0.085). There was no evidence of a statistically significant change in SF-8^TM^ scores over time. Participants consistently reported positive satisfaction scores with RWC initiatives.

Conclusions

Dedicated RWC, with input from both faculty and resident physicians, improved wellness during residency training with a high degree of participant satisfaction. Such programs are needed to support resident physicians during their training.

## Introduction

It is well-established that resident physician training, or “residency,” is a challenging period in the career path of physicians. Many authors have characterized the morbidity of a struggling physician in terms of “burnout,” a quantifiable psychologic syndrome composed of emotional exhaustion, depersonalization, and reduced personal accomplishment [1]. Others have highlighted alarming rates of depression [2-3], suicide [4], as well as relationship peril [5] and sexual dysfunction [6], attributed to residency training. Resident burnout is pervasive across various resident subspecialties and has been linked to suboptimal patient care and medical error [7].

In the recent past, many hypothesized that burnout issues were born from excessive resident duty hours. In response, duty hours were shortened and regulated in the United States [8], Europe [9], and Canada [10] in 2003, 2009, and 2010, respectively. The subsequent analysis of duty hours reform, however, revealed no improvement in sleep hours, work hours, medication errors, or depression [11]. Moreover, despite its good intentions, duty hours reform has also been met with criticism over concern for adequate training experience and compromised patient care [12].

Recently, some authors have advocated for a new approach to addressing resident burnout: dedicated wellness curricula during residency training [7,13]. Resident wellness curricula (RWC) aim to add support to residents rather than detract from hours of experience. Ideally, the support offered in an RWC should include initiatives that are both active and passive, preventive and responsive, and address the spheres of physical, mental, educational, social, financial, and lifestyle wellness [7,14-15]. Given that RWC are a relatively new concept, there is currently a lack of literature demonstrating efficacy with validated instruments. To date, most of the evidence for wellness initiatives is derived from mindfulness education sessions initiated by the researchers themselves [16-17]. We hypothesized that a robust RWC, with parallel input from both faculty and the resident body, would lead to a significant improvement in resident wellness measurable with a validated scoring tool, the Brief Resident Wellness Profile (BRWP) [18] and/or the SF-8TM Health Survey [19].

## Materials and methods

In Canada, dedicated Emergency Medicine residency training is accredited by the Royal College of Physicians and Surgeons. To achieve fellowship designation, trainees must complete a five-year, post-graduate residency program at a designated university. This training includes an initial basic clinical training year with mixed rotations through surgery, obstetrics, internal medicine, anesthesia, and so forth. Subsequent training years include rotations in intensive care with increasing time spent in the Emergency Department. Senior residents in years four and five practice almost exclusively in emergency medicine.

This study was reviewed and approved by the Research Ethics Office at the University of Alberta (Pro00024292). Subsequent to ethics approval, Royal College resident physicians in the Department of Emergency Medicine were hand-delivered surveys at scheduled academic events in 2009, 2010, and 2011. These dates corresponded to a baseline (no RWC) assessment, one year post-exposure to a faculty-derived RWC (F-RWC), and one year post-exposure to parallel faculty and resident-derived RWC (R-RWC). Of note, the 2009 and 2010 groups were identical, however, owing to both graduation and recruitment, the 2011 group had some new participants and slightly larger sample size. Residents not in attendance had surveys distributed to their individual hospital mailboxes. Survey completion was voluntary, and consent was implied with the confidential return of sealed surveys to the Program Administrator. Participant information was not linked across all three time points. Surveys were held in a locked cabinet in the Department of Emergency Medicine until the time of data analysis after 2011.

Survey packages included the Brief Resident Wellness Profile (BRWP) with faces scale [18], the SF-8^TM^ Health Survey [19], and a demographics section. The 2011 cohort also received a satisfaction survey composed of a seven-point Likert scale for each of the RWC initiatives completed in the year prior. In addition, participants were invited to leave comments in a text box to further describe their degree of satisfaction with the program.

The F-RWC was developed and implemented by two Emergency Physicians who were educators in the resident training program. The F-RWC consisted of the following: 1) biannual, confidential, one-on-one meetings between each resident and a dedicated faculty wellness mentor; and 2) wellness-related lectures added to mandatory academic rounds. The goal of the one-on-one meetings was to identify and intervene if symptoms of burnout and depression, problematic substance use, relationship issues, and financial concerns and/or challenges in the hospital setting were present. Each session was scheduled for one hour and residents were able to schedule more meetings with the wellness mentor if needed or desired. The wellness mentors were also available throughout the year for urgent wellness-related concerns. Grand Rounds topics included fitness, healthy food preparation, and financial planning.

The R-RWC was developed and implemented by a senior resident in the Department of Emergency Medicine as part of an academic research initiative. The R-RWC was targeted to specific wellness domains and consisted of the following:

1) Educational Wellness: post-graduate year (PGY)-1 “on-call survival booklet,” PGY-2 exam binders to streamline preparation for the Medical Council of Canada Qualifying Exam Part II, PGY-3 primer booklet for the eight-week intensive care unit (ICU) rotation, and a toolkit for all residents, including drug reference booklet, reference cards, and pocket light.

2) Social Wellness: monthly or semi-monthly organized outings to local professional sports teams, live music, or dinner engagements.

3) Mental Wellness: monthly new music playlists for exercise or study, wellness seminar at the annual resident retreat, Christmas gift donation drive, blood donation drive, and Remembrance day perspective material.

4) Financial Wellness: access to income tax software, funding for travel to the annual resident retreat, and coupons to local shops.

5) Lifestyle Wellness: healthy and easy recipes for home-cooked meals, a non-medical library with recent novels and magazines.

6) Physical Wellness: 24-hour access to complimentary bottled water and sports drinks in the resident lounge.

Data analysis included descriptive statistics (e.g., mean, standard deviation (SD), frequencies, and percents) for demographic variables and outcomes by year. Mixed-effects linear regression models were used to assess the effect of year on continuous outcomes (random effect for the subject as the same subjects were in the first two years). Additional explanatory variables (age, sex, marital status, debt amount, debt indicator, and moonlight) were also added to examine the adjusted effect of year. Fisher’s exact (FE) test assessed the association between faces scores and year. P-value (p) less than 0.05 was considered statistically significant. Analyses were performed in R Core Team (2016) [20].

## Results

Resident demographics

Anonymized information on residents in the program is depicted in Table 1. There was a total of 58 participants in this study with a slight predominance of males (62%). The ages were similar among groups with a mean age of 30.65, 31.65, and 30.17 years for 2009, 2010, and 2011, respectively. Other demographic variables, including marital status, children, debt, and frequency of moonlighting shifts, also remained similar over the three time points (Table 1). All subjects were emergency medicine residents from the University of Alberta, Canada.

**Table 1:** Demographic information by cohort year

	2009	2010	2011
Sex			
Male	12	12	12
Female	5	5	11
Missing	0	0	1
Total	17	17	24
Age			
Mean (standard deviation)	30.65 (2.12)	31.65 (2.12)	30.17 (2.94)
Marital Status			
Married or Common Law	6	6	9
Dating	7	7	4
Divorced or Widowed	0	0	0
Single	4	4	10
Missing	0	0	1
Children			
Yes	0	0	2
No	17	17	22
Missing	0	0	0
Debt Amount (CAD)			
< $10,000 in debt	3	3	3
$10,000-$99,999	8	7	10
$100,000-$199,999	6	7	5
≥$200,000	0	0	4
Moonlight			
Never	6	5	6
< once per month	2	1	3
~ one shift per month	6	8	6
2-4 shifts per month	3	3	6
>4 shifts per month	0	0	1
Missing	0	0	1

Survey response

The response rates for surveys completed in 2009, 2010, and 2011 were 89% (n=17), 100% (n=17), and 83% (n=24), respectively.

Wellness scores

**Figure 1:**
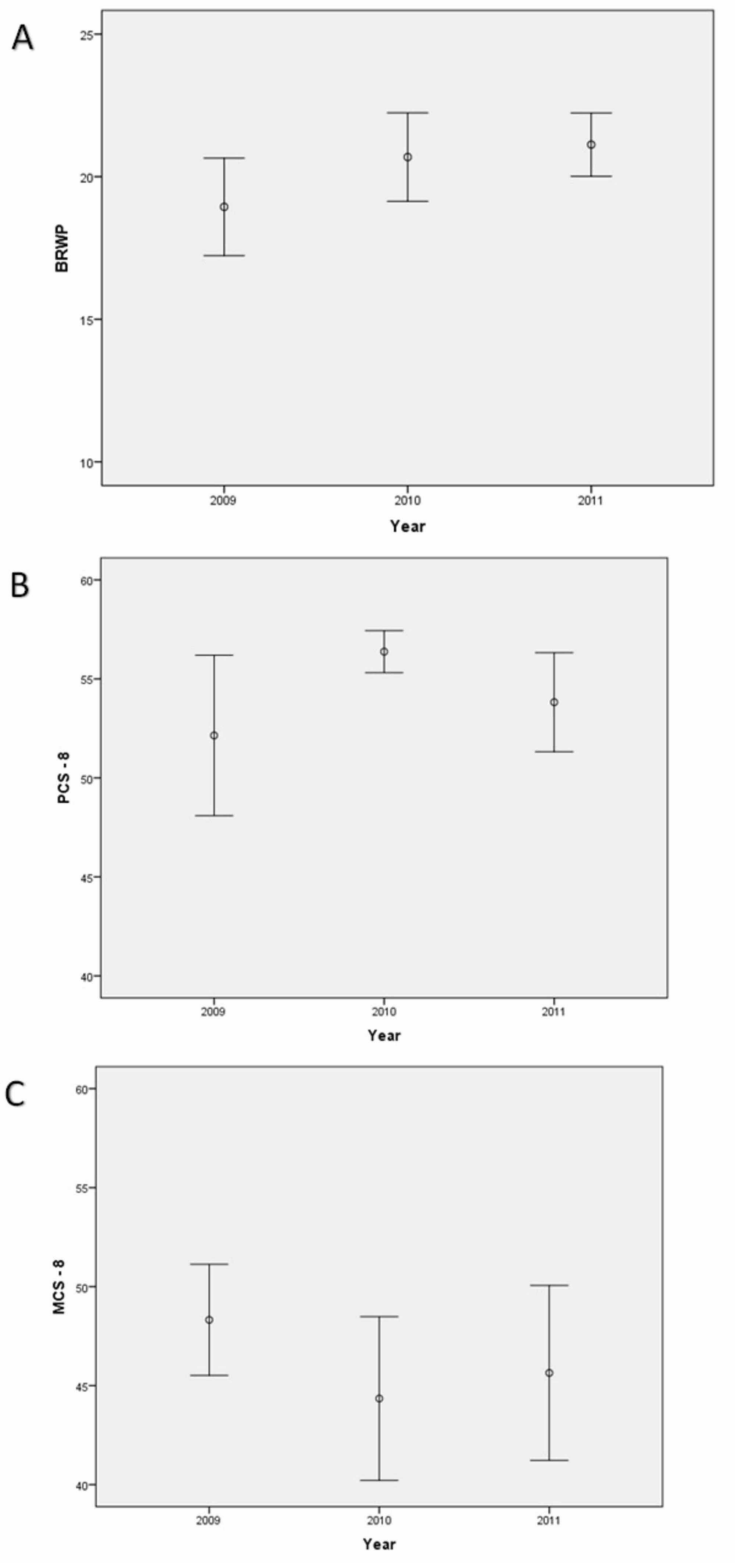
Wellness scores A) Scores from the Brief Resident Wellness Profile (BRWP; error bars represent mean and 95% confidence interval for each year). There was a significant improvement over time from 2009 to 2011 (p=0.024); B) Scores from the SF-8 health survey, Physical Component Summary (PCS-8; p=0.174); C) Scores from the SF-8 health survey, Mental Component Summary (MCS-8; p=0.392).

Mean scores for the Brief Resident Wellness Profile (BRWP) in 2009 (baseline), 2010 (one year after Faculty-derived Resident Wellness Curriculum; F-RWC), and 2011 (one year after combined F-RWC and Resident-derived Resident Wellness Curriculum; R-RWC) were 18.94, 20.69, and 21.12 (p=0.024), respectively, demonstrating a significant improvement in resident wellness after exposure to the combined RWC from 2009 to 2011 (Figure 1A). Even when adjusted for other factors in a multivariable mixed model (see the methods section), the BRWP remained different over the years (p=0.011). There was no evidence of a statistically significant change over time measured by the SF-8TM Health Survey (Figures 1B-1C) in either the Physical Component Summary (PCS-8; KW test, p=0.174) or the Mental Component Summary (MCS-8; KW test, p=0.392).

Faces scores

**Figure 2:**
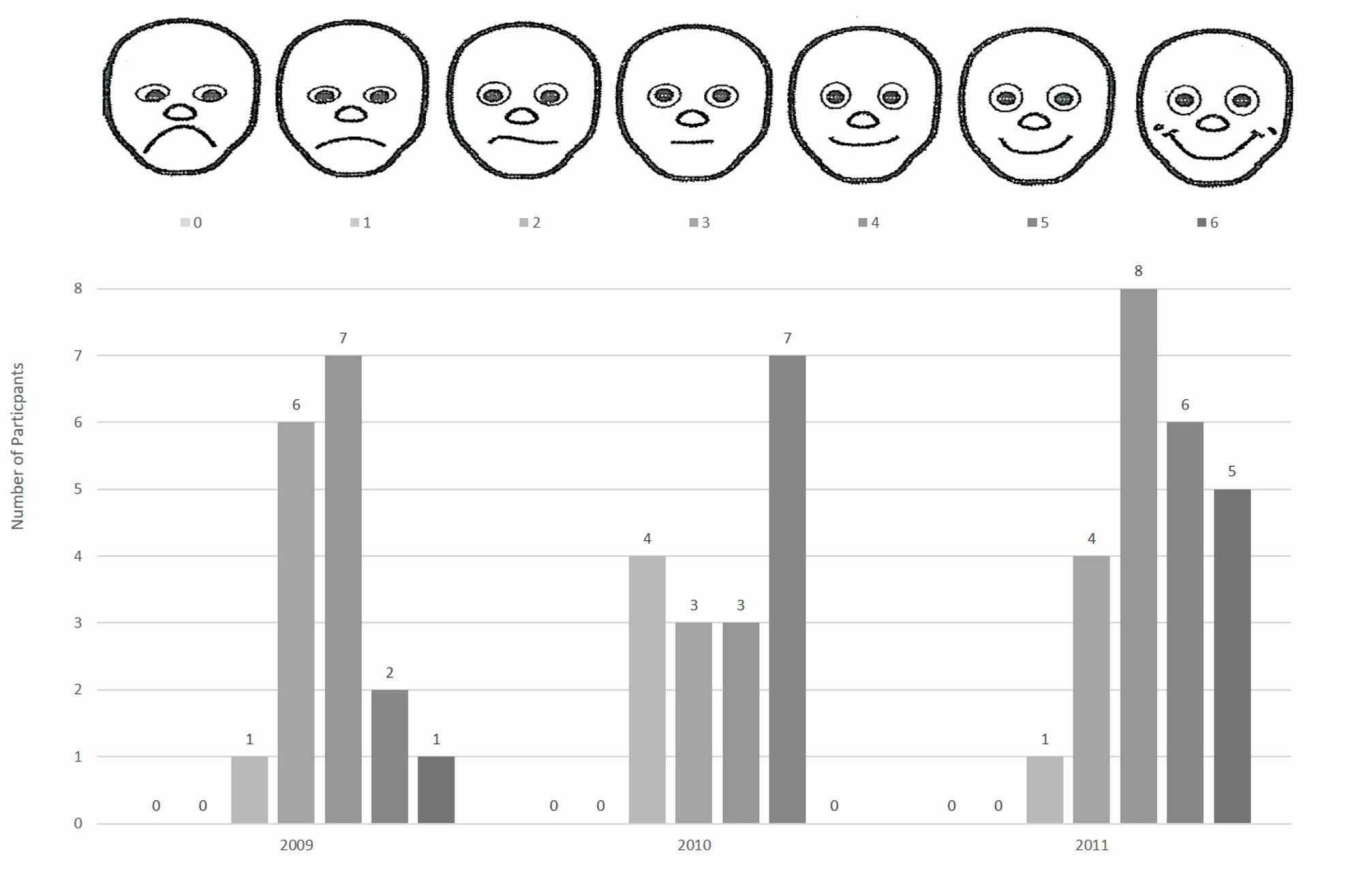
Faces scores Faces scores (range from most unhappy face =0, to happiest face =6). The Fisher exact test showed improvement from 3.765 to 4.417 (from 2009 to 2011) but did not reach statistical significance (p=0.084). Faces illustration from Keim et al. 2006 [18].

This subset of the BRWP tended to be higher for 2011 than 2009 (Figure 2), suggesting a trend toward improvement, however, there was no evidence of an association between the faces score and year (FE test, p=0.085).

Program evaluation

**Figure 3:**
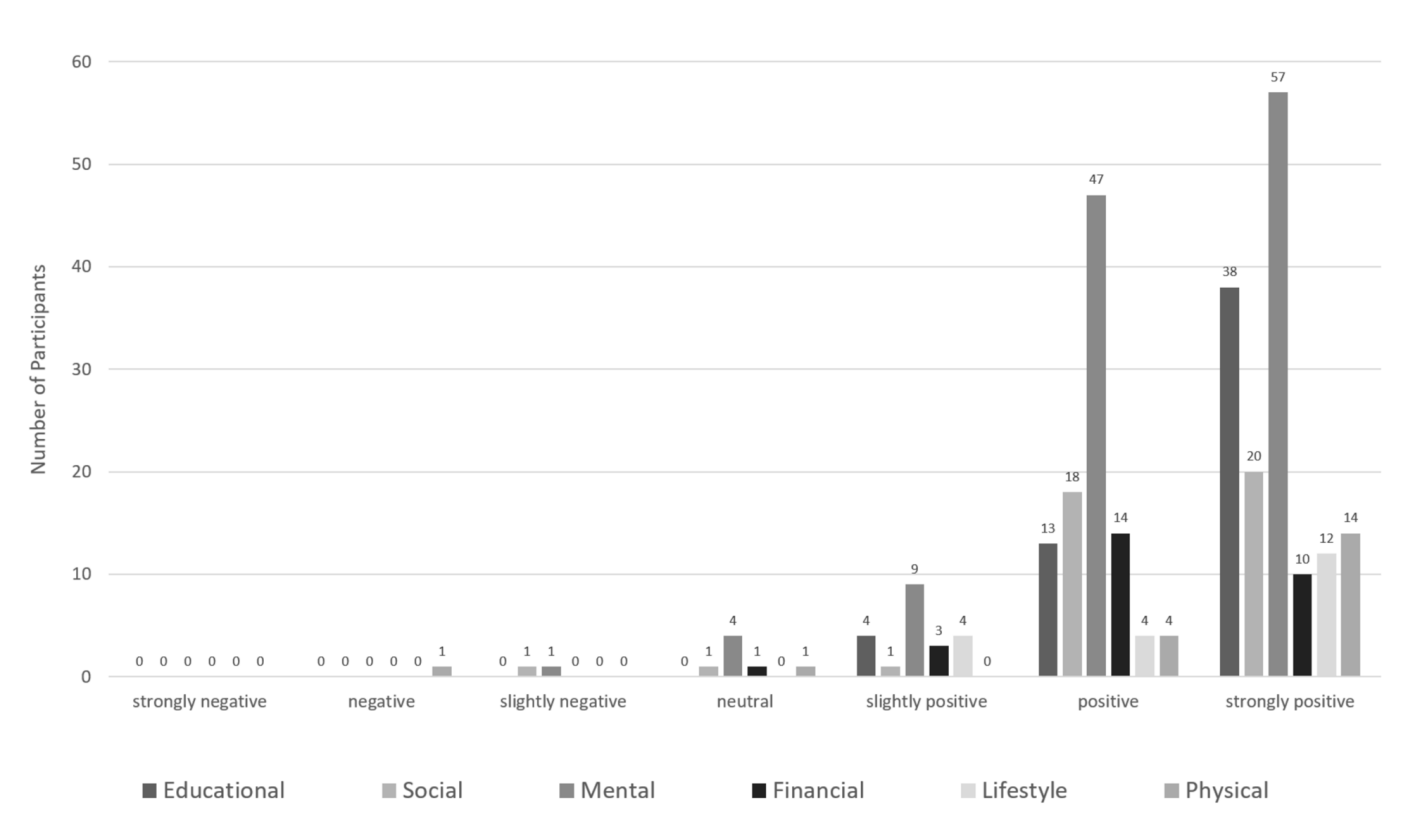
Resident satisfaction with the Resident-derived Resident Wellness Curriculum (R-RWC)

Resident participation and satisfaction with the R-RWC were assessed in 2011 using a seven-point Likert scale (Figure 3). Each survey organized and listed R-RWC interventions under the five domains of wellness (educational, social, mental, financial, lifestyle, and physical) to enhance recall. There were a total of 282 participants at various R-RWC events, and 272/282 (96%) indicated a slightly positive, positive, or strongly positive experience. Further, 151/282 (54%) reported a strongly positive satisfaction score. Mental wellness initiatives (see the methods section) were the best-attended events.

## Discussion

Residency training is a stressful period for learners, and recent literature demonstrates the consequences of these risks and the steps taken to address them. In a landmark case, the death of Libby Zion in 1984 revealed a potential link between resident burnout and patient safety [21]. Over the next 20 years, researchers consistently published alarming rates of burnout [22-26], depression [2-3], morbidity [27], and mortality [4] among resident physicians. Many hypothesized that the main culprit was excessive duty hours during residency training. However, multinational regulations of duty hours [8-10] in the 2000s failed to alleviate the substantial risks associated with training [28] and were subsequently met with widespread criticism from educators [12].

Since modification of duty hours alone has not eliminated the development of burnout, additional interventions are required to address this issue. The current study adds to a growing body of literature suggesting that dedicated wellness training is beneficial to resident physicians and the patients they treat [7,12,16]. To our knowledge, this is the first study to investigate the efficiency and satisfaction of a multi-faceted RWC using a validated tool tailored specifically for resident physicians. Unique to our program is the combination of both faculty and resident input, the targeting of multiple wellness domains, and the use of both active and passive interventions.

The key finding in our study is that the combination of F-RWC and R-RWC over a two-year period led to a significant benefit in resident wellness as measured by the BRWP. During this period, no other major interventions were adopted or implemented that could explain these changes. Moreover, duty hours and residency rotations were unchanged. Of note, no evidence of a statistically significant benefit seen with F-RWC alone that may suggest that input from the resident population (the target audience) was critically important. However, this outcome could also be the result of the relatively low sample size.

There was no evidence of a statistical benefit seen in physical and/or mental health as measured by the SF-8TM Health Survey. This instrument provides a generic measure of health that is not specific to any particular population. Conversely, resident physicians are a niche population, of similar age, with stressors that are unique to this profession. This is likely the reason that a benefit was seen with a tailored resident wellness instrument (BRWP) as opposed to the non-specific SF-8TM.

A secondary finding in our analysis was the high level of resident satisfaction with the program. This indicates that not only does our RWC improve wellness, but it is also well-liked by the target audience. Written comments from residents were consistently positive. Some examples from the satisfaction survey included: “Fantastic, made me feel like someone cared about my success both at work and away from work,” “Prevents the degree of separation that naturally occurs between staff and residents,” and “I think it is very positive, especially for new residents joining the group for support and sense of community.”

The strengths of our study include the multi-faceted nature of the program, the use of published and validated instruments to measure resident physician wellness (BRWP) and general health (SF-8TM), the measurement of wellness at three different time points, and the high survey response rate. There are also some limitations to this study. First, the study population is small (total of 58 participants) and only includes residents in the Emergency Medicine training program from a single institution. Second, the cross-sectional format of this study means that cohorts from 2009, 2010, and 2011 had different resident populations with variable exposures to our RWC. This is due to resident turnover, as each year graduating residents become staff, and graduating medical students begin their residency training. Third, owing to small cohort sizes, there were not enough participants to include a parallel control group over the three time points. Finally, due to the need for anonymity, details about non-responders and individual changes over time were not able to be analyzed.

In conclusion, this small, single-center study shows that dedicated, multi-faceted RWC with input from both faculty and resident physicians can provide a significant wellness benefit to a resident population that strongly supports the program. Future research is needed to assess similar curricula in larger resident populations, in other subspecialties, and at other institutions. These programs are effective, well-liked, well-attended, and are designed to minimally interfere with formal resident education or duty hours.

## Conclusions

Residency training is a challenging period in the development of future physicians. For some, the demands and stressors of training can lead to burnout or worse. To date, putative solutions to this issue, such as duty hours reform, have shown to be ineffective. In response, a novel approach is an introduction of dedicated Resident Wellness Curricula (RWC) to residency training. Here, dedicated RWC with input from both faculty and resident physicians improved wellness during residency training with a high degree of participant satisfaction. Such programs are needed to support resident physicians during their training.
